# Re-visiting the Protamine-2 locus: deletion, but not haploinsufficiency, renders male mice infertile

**DOI:** 10.1038/srep36764

**Published:** 2016-11-11

**Authors:** Simon Schneider, Melanie Balbach, J F J Jan F. Jikeli, Daniela Fietz, Daniel Nettersheim, Sina Jostes, Rovenna Schmidt, Monika Kressin, Martin Bergmann, Dagmar Wachten, Klaus Steger, Hubert Schorle

**Affiliations:** 1 Institute of Pathology, Department of Developmental Pathology, University of Bonn Medical School, Bonn, Germany; 2 Minerva Max Planck Research Group - Molecular Physiology, Center of Advanced European Studies and Research, Bonn, Germany; 3 Institute of Veterinary-Anatomy, -Histology and -Embryology, Justus-Liebig University, Giessen, Germany; 4 Department of Urology, Pediatric Urology and Andrology, Section Molecular Andrology, Biomedical Research Center of the Justus-Liebig University, Giessen, Germany

## Abstract

Protamines are arginine-rich DNA-binding proteins that replace histones in elongating spermatids. This leads to hypercondensation of chromatin and ensures physiological sperm morphology, thereby protecting DNA integrity. In mice and humans, two protamines, protamine-1 (*Prm1*) and protamine-2 (*Prm2*) are expressed in a species-specific ratio. In humans, alterations of this PRM1/PRM2 ratio is associated with subfertility. By applying CRISPR/Cas9 mediated gene-editing in oocytes, we established *Prm2*-deficient mice. Surprisingly, heterozygous males remained fertile with sperm displaying normal head morphology and motility. In *Prm2*-deficient sperm, however, DNA-hypercondensation and acrosome formation was severely impaired. Further, the sperm displayed severe membrane defects resulting in immotility. Thus, lack of *Prm2* leads not only to impaired histone to protamine exchange and disturbed DNA-hypercondensation, but also to severe membrane defects resulting in immotility. Interestingly, previous attempts using a regular gene-targeting approach failed to establish *Prm2*-deficient mice. This was due to the fact that already chimeric animals generated with *Prm2*^+/−^ ES cells were sterile. However, the *Prm2*-deficient mouse lines established here clearly demonstrate that mice tolerate loss of one *Prm2* allele. As such they present an ideal model for further studies on protamine function and chromatin organization in murine sperm.

The production of mature sperm starts with diploid spermatogonial stem cells, which undergo mitotic and meiotic division and finally differentiate into haploid spermatids. During spermiogenesis, round spermatids develop into elongated spermatids, with a species-specific head morphology. In developing male germ cells, DNA-binding histones are gradually replaced by testis-specific histone variants, transition proteins, and finally protamines, resulting in a hypercondensation of the paternal haploid genome^1^.

Protamines are rich in positively charged arginine residues, mediating strong binding to the minor groove of the negatively charged DNA^2^. In addition, cysteine residues form intra- and intermolecular disulfide bonds, thereby stabilizing the DNA-protamine complex^2^,^3^. The sperm-protamine complexes form toroids, leading to an at least 6-fold denser compaction compared to histone-bound DNA of mitotic chromosomes^4^. Each toroid comprises around 60 kb of DNA^5^. As a consequence, this hypercondensation in elongated spermatids causes a complete silencing of transcription and protects the genome from environmental insults^6^,^7^.

Although many mammals express only a single protamine gene, humans and rodents encode two protamine genes^8^,^9^,^10^. In mice and humans, the genes encoding protamine-1 (*Prm1*), protamine-2 (*Prm2*), and transition protein-2 (*Tnp2*) form a conserved and tightly regulated gene cluster located on chromosome 16^11^,^12^. PRM1 is translated as a mature protein, whereas PRM2 is translated as a precursor that matures by cleavage of its N-terminal part^8^,^13^,^14^. Interestingly, PRM1 and PRM2 are translated in a species-specific ratio. In humans, both isoforms are present in approximately equal amounts, whereas in mice, PRM2 is more abundant, accounting for approx. 65% of total protamine^15^. In some species, such as boar, bull, and rat, PRM2 is virtually absent due to mutations within the coding or promoter region^16^,^17^. In humans, aberrant protamine ratios are associated with subfertility and decreased DNA integrity^18^,^19^,^20^,^21^,^22^,^23^,^24^,^25^. Furthermore, altered protamine ratios are associated with decreased fertilization rates in men participating in assisted reproduction technology (ART) programs^26^,^27^,^28^. Using a standard gene-targeting approach, both murine protamine genes have been deleted^29^,^30^. However, chimeras generated by injecting ES cells heterozygous for one allele of either protamine gene resulted in infertility. Sperm displayed abnormal head morphology, disturbed DNA integrity, and decreased motility. As a consequence, *Prm2*-deficient mouse lines could not be established, hampering further experiments dealing with Protamine function.

In this study, we used the CRISPR/Cas9 technology to generate *Prm2*-deficient mice. Application of this genome editing tool in murine zygotes allows to precisely and efficiently edit genes without disturbing the overall exon-intron gene structure or deleting regulatory DNA sequences^31^. We demonstrate that mice heterozygous for *Prm2* are fertile with normal sperm morphology and motility, whereas *Prm2*-null mice are infertile due to a complete loss of sperm motility and abnormal sperm head morphology.

## Results

### CRISPR/Cas9-mediated generation of *Prm2*-deficient mice

Previously, it was reported that chimeric mice generated by injecting *Prm2*^+/−^ ES cells were sterile^29^. In general, ES cell lines are of male sex and mediate sex-reversal when injected into female host blastocysts. Thus, a mouse line harboring a mutation affecting the male germline, like the *Prm2*-heterozygosity, cannot be established by this technology, precluding detailed research of protamine biology. Since protamines are only required for male germ cell development, establishment of *Prm2*-deficient mouse lines should be possible from female founder animals. Therefore, we decided to apply CRISPR/Cas9-mediated gene-editing in murine zygotes to generate *Prm2*-deficient mice^31^.

**Figure 1:**
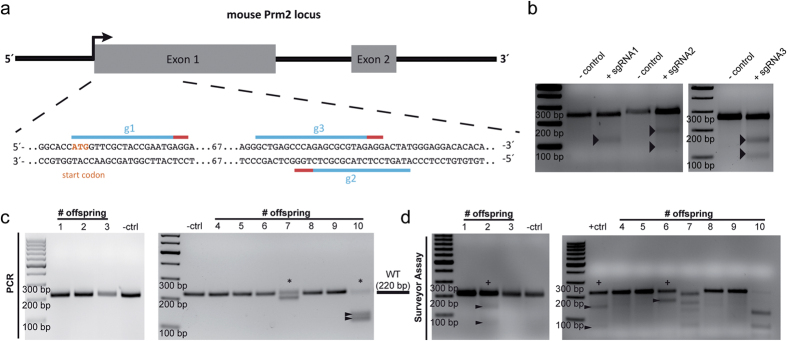
CRISPR/Cas9-mediated generation of Prm2-deficient mice. **(a)** Schematic representation of the *Prm2* gene locus and targeting sites of designed gRNAs. **(b)** Surveyor Assay. Intended target sites of gRNAs were amplified by PCR from genomic DNA of gene-edited E14TG2a ES cells. Wildtype and gene-edited PCR products were denaturated and re-annealed. Surveyor nuclease mediated cleavage of mismatched PCR products (highlighted by arrowheads) confirmed functionality of gRNAs. **(c,d)** Genotyping of offsprings by PCR and Surveyor assay identified 4/10 animals to be mutant as indicated by asterisks (PCR) and pluses (Surveyor).

The murine *Prm2* gene consists of two exons, encoding the 106 amino acids-long precursor protein (Fig. 1a). We derived three guide RNAs (gRNAs) targeting the first exon close to the translational start site (Fig. 1a). First, the gRNAs were validated in murine ES cells. CRISPR/Cas9 induces double-strand breaks, leading to error-prone non-homologous end-joining mediated repair^32^. This results in formation of small insertions or deletions (indels), which can be detected by the ‘Surveyor nuclease’, resulting in cleavage of PCR products. The gene-edited *Prm2*-coding region was amplified by PCR and subjected to the Surveyor assay^33^, which confirmed the functionality of derived gRNAs, as indicated by cleavage of the PCR products (Fig. 1b, cleavage products highlighted by arrowheads).

To edit the *Prm2* gene in murine zygotes, we microinjected *in vitro* transcribed single-gRNAs (sgRNAs) and *Cas9* mRNA into the cytoplasm. A combination of sgRNAs 1 and 2 or sgRNAs 1 and 3 was applied to induce deletions of approximately 100 bp. From 336 injected zygotes, 245 (72.9%) survived (Table 1); 82 blastocysts were transferred and 10 viable pups were born (Table 1). Genotyping by PCR and Surveyor assay identified 4/10 pups carrying CRISPR/Cas9-induced mutations within the *Prm2* locus (Fig. 1c,d). Of those, three animals (# 2, 6, 7) contained small indels, indicating that one sgRNA had catalyzed gene-editing. One female (#10) harbored the intended deletion of approximately 100 bp (Fig. 1c). Sequence analysis of genomic DNA from #10 revealed that the CRISPR/Cas9-mediated gene-editing resulted in four different alleles. Besides the wildtype allele, three alleles with deletions of 97 bp, 101 bp, and 103 bp were obtained. This suggests that gene-editing took place at the 2-cell-stage. Alleles with deletions of 97 bp and 101 bp were separated by back-crossing with C57BL/6 wildtype mice.

### Haploinsufficiency of *Prm2* does not affect male fertility

**Figure 2:**
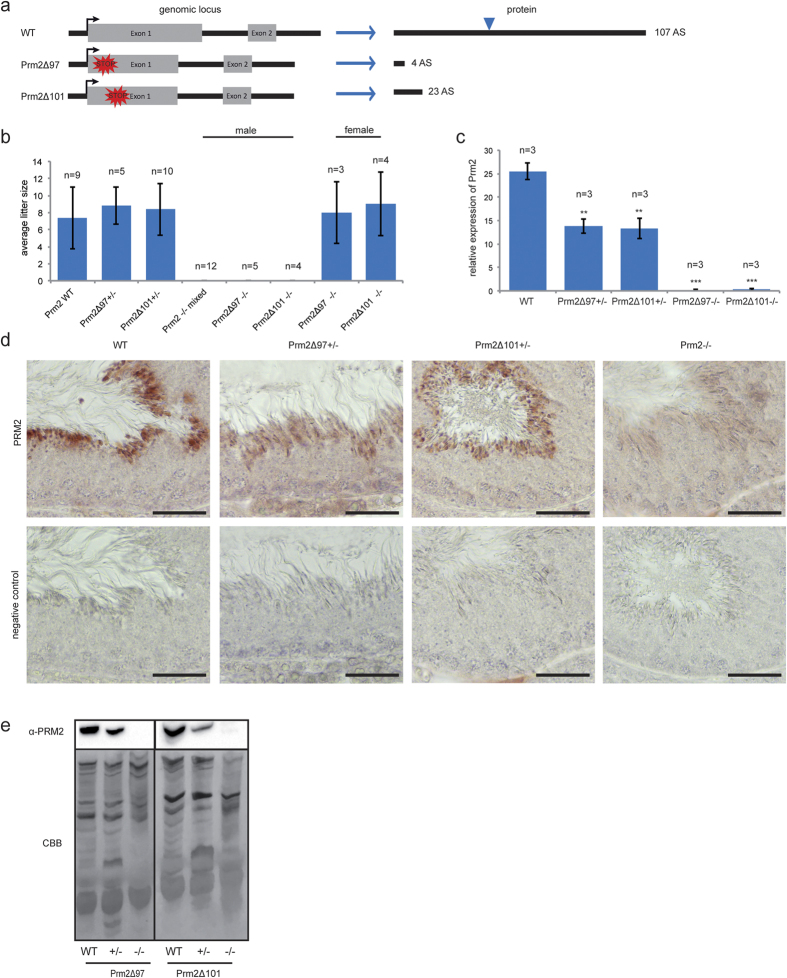
Knockout validation and fertility analysis. **(a)** Schematic representation of established *Prm2*-deficient mouse lines Prm2Δ^97bp^ and Prm2Δ^101bp^. Blue triangle marks cleavage site within the premature PRM2 protein. **(b)** Mating statistics of *Prm2*-deficient males and females. Successful mating of Prm2^−/−^ males with wildtype females was indicated by presence of a vaginal plug at 0.5 dpc. **(c)** Relative expression of *Prm2* mRNA in murine testis of wildtype, *Prm2*^+/−^, and *Prm2*^−/−^ mice measured by qRT-PCR. **(d)** Immunohistochemical staining of PRM2 on testicular sections of wildtype, *Prm2*^+/−^ and *Prm2*^−/−^ mice. Scale bar = 100 μm. **(e)** Immunoblot against PRM2 following acid urea gel electrophoresis of basic nuclear proteins isolated from epididymal sperm of Prm2Δ^97bp^ or Prm2Δ^101bp^ mice. Of note, the amount of protein loaded from *Prm2*-deficient samples were doubled compared to samples from wildtype and heterozygous animals. Coomassie-Brilliant-Blue staining (CBB) served as loading control. The antibody detects mature PRM2 as well as its precursor forms (pP2-a, pP2-b).

Two mouse lines, Prm2Δ^97^ (harboring a 97 bp deletion) and Prm2Δ^101^ (harboring a 101 bp deletion), were established. On both alleles, a frameshift was generated, resulting in nonsense transcripts with a premature stop codon and deletion of *Prm2* (Fig. 2a). In contrast to earlier reports^29^, male mice heterozygous for either Prm2Δ^97^ or Prm2Δ^101^ were fertile and sired offspring. Matings of males heterozygous for either Prm2Δ^97^ or Prm2Δ^101^ produced an average litter size of 8.6 (Fig. 2b). There was no significant difference in the average litter size among the two mouse lines (8.8 vs. 8.4). This is comparable to published data for wildtype mice with a mean litter size of 6.2 (9.5 in the plateau period of reproduction)^34^,^35^ and our wildtype matings with an average litter size of 7.3. In contrast, male mice homozygous for either Prm2Δ^97^ or Prm2Δ^101^ were not able to reproduce (Fig. 2b), whereas female mice homozygous for either Prm2Δ^97^ or Prm2Δ^101^ were fertile and sired offspring with an average litter size of 8.5, when mated with heterozygous males (Fig. 2b). These results clearly indicate that loss of one allele of *Prm2* can be tolerated, whereas complete lack of *Prm2* results in male sterility.

To validate that gene-editing generated a *Prm2* loss-of-function allele, we analyzed full-length *Prm2* RNA levels. Since *Prm2* is expressed during spermiogenesis in step 7–15 spermatids, qRT-PCR was performed on RNA isolated from murine testis^36^. In heterozygous animals, relative *Prm2* mRNA expression levels were reduced by approximately 50% compared to wildtype animals (Fig. 2c). Sperm from males homozygous for either mutation did not show any *Prm2* signal, indicating that the gene-editing led to successful deletion of the *Prm2* gene (Fig. 2c).

Immunohistochemical staining (IHC) using an anti-PRM2 antibody on testicular tissue sections detected the protein in spermatids at stage VIII of the seminiferous epithelial cycle of both, wildtype and *Prm2*^+/−^ mice (Fig. 2d). The staining appeared less intense in heterozygous mice, suggesting a reduction in protein level due to decreased mRNA levels. In accordance with qRT-PCR data, sections from *Prm2*^−/−^ testes were negative for PRM2 at all stages of the mouse seminiferous epithelial cycle, further confirming the deletion of the gene (Fig. 2d, right).

Western blot analysis of basic nuclear proteins isolated from epididymal sperm was performed to determine protein levels of PRM2. Males heterozygous for either Prm2Δ^97^ or Prm2Δ^101^ displayed an approximately 50% decrease of mature PRM2 compared to wildtype controls (Fig. 2e). In *Prm2*^−/−^ mice, mature PRM2 protein was not detectable (Fig. 2e).

### *Prm2*-deficient sperm display severe morphological defects

**Figure 3:**
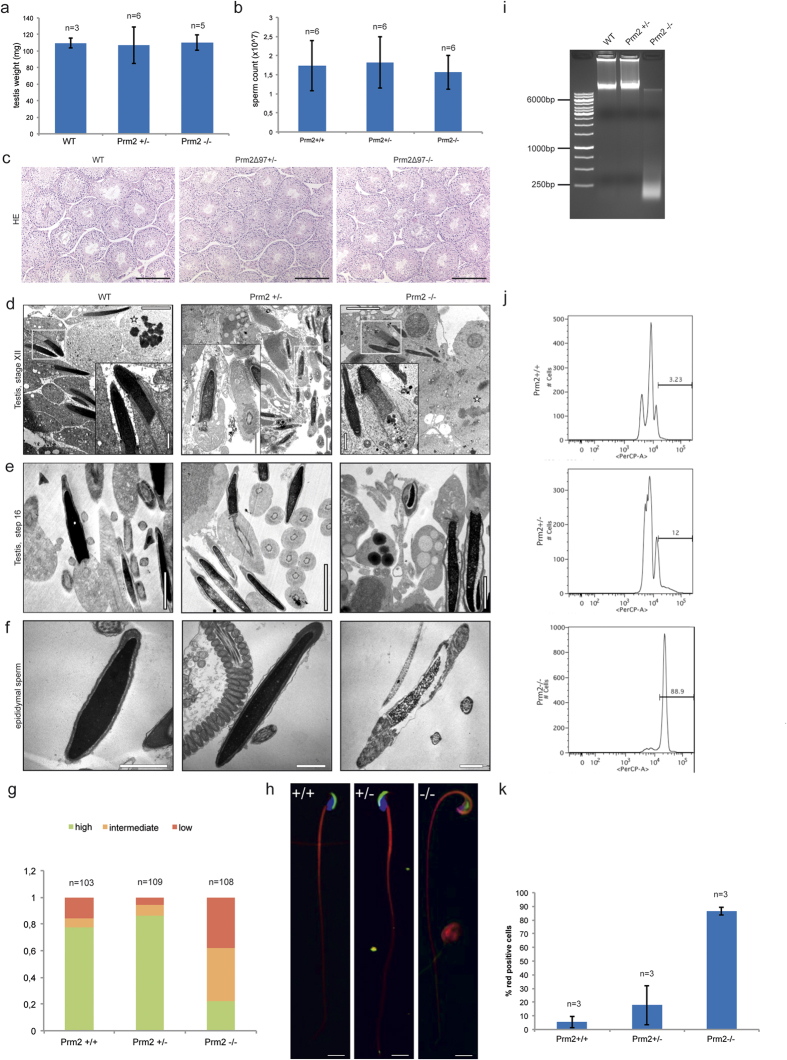
Morphological testes and sperm analysis. **(a)** Testes weight. **(b)** Epididymal sperm count of *Prm2*^+/+^, *Prm2*^+/−^, and *Prm2*^−/−^ mice. **(c)** HE staining of testicular sections from wildtype, *Prm2*^+/−^ and *Prm2*^−/−^ mice. Scale bar = 200 μm. **(d)** Transmission electron micrographs (TEM) of stage XII seminiferous epithelium as indicated by meiotic spermatocytes (stars) showing step 12 elongating spermatids. Scale bar = 5 μm. Inset: enlargement of rectangle, scale bar = 1 μm. Note homogeneously condensed chromatin in wildtype in contrast to heterogeneously condensed fine-grained chromatin in the *Prm2*-deficient mutant, whereas formation of the acrosome and manchette as well as head shape did not differ. **(e)** TEMs of step 16 elongated spermatids prior to sperm release. Scale bar = 1 μm. **(f)** TEMs of epididymal sperm. Note nuclear matrix alteration, detachment of acrosome (arrow) and attachment of the flagellum to the head (arrowhead) in knockout sperm. Scale bar = 1 μm. **(g)** Quantification of epididymal sperm chromatin integrity. **(h)** *Prm2*^−/−^ but not *Prm2*^+/−^ sperm showed morphological defects in the acrosome and head-tail conjunction. The acrosome was labeled using PNA-FITC (green), DNA using DAPI (blue), and the flagellum using MitoTracker (red). Scale bar = 10 μm. **(i)** Agarose gel electrophoresis of sperm genomic DNA. **(j,k)** Sperm damage analysis referring to SCSA.

Because *Prm2*^+/−^ males were fertile, we were able to breed *Prm2*^−/−^ animals and analyze the effects of loss of *Prm2* in more detail. Since testis weight (Fig. 3a), epididymal sperm count (Fig. 3b), and testis morphology was not changed in *Prm2*^+/−^ and *Prm2*^−/−^ animals compared to wildtype (Fig. 3c), we concluded that spermatogenesis is not affected. To investigate why *Prm2*-deficient male mice are infertile, we analyzed the morphology of testicular spermatids and epididymal sperm.

Electron microscopy of *Prm2*-deficient testicular spermatids revealed impaired nuclear matrix morphology, exhibiting a fine-grained, sometimes coarse-grained texture (Fig. 3d). Anomalies in chromatin condensation were obvious from step 12 spermatids onward. Until sperm release, the heterogeneity in chromatin packaging, including vesiculation, became more pronounced (Fig. 3e). However, sperm head elongation and shaping were not affected in testicular spermatids (Fig. 3d,e).

Next, epididymal sperm were co-stained with PNA-FITC to label the acrosome, MitoTracker to label mitochondria in the midpiece of the flagellum, and DAPI to label the DNA in the sperm head (Fig. 3h). Wildtype mice and mice heterozygous for Prm2Δ^97^ or Prm2Δ^101^ showed the typical sickle-shaped head with the acrosome lining the sperm head, whereas *Prm2*-deficient sperm lacked the acrosome and the midpiece of the flagellum was wrapped around the sperm head (Fig. 3h). The majority of *Prm2*-deficient sperm showed this phenotype, which was confirmed by electron microscopy (Fig. 3f). On the ultrastructural level, *Prm2*-deficient sperm displayed detachment of the acrosome at the acrosome-nuclear interface, giving rise to an abnormally shaped head with an undulating plasma membrane (Fig. 3f). In line with observations from testicular spermatids, chromatin integrity was highly disturbed in epididymal sperm (Fig. 3f). Quantification of chromatin integrity revealed that around 80% of *Prm2*-deficient sperm showed intermediate to low chromatin density. No difference was observed between wildtype and *Prm2*^+/−^ animals with around 80% of sperm showing high chromatin integrity (Fig. 3g). Thus, loss of *Prm2* not only affects DNA integrity in the sperm head, but also bending of the flagellum and anchorage of the acrosome. To analyze whether a loss of DNA integrity makes sperm more susceptible to DNA damage, we subjected genomic DNA from sperm to agarose gel electrophoresis. While a band indicative of high molecular weight DNA suggested little to no DNA damage in sperm of *Prm2*^+/+^ and *Prm2*^+/−^ animals, the majority of sperm DNA from *Prm2*-deficient mice was strongly degraded into fragments of around 250 bp in size (Fig. 3i). Next, sperm were treated with a strong acid to induce denaturation of DNA, followed by staining with acridine orange and FACS analysis, referring to the sperm chromatin structure assay (SCSA). Intercalation of acridine orange into intact, double-stranded DNA results in emission of green fluorescence, whereas incorporation into damaged, single-stranded DNA results in red fluorescence. In accordance with the results from the agarose gels, red fluorescence intensity was strongly increased in *Prm2*-deficient sperm compared to *Prm2*^+/+^ and *Prm2*^+/−^ animals (Fig. 3j). In total, more than 80% of *Prm2*-deficient sperm cells showed intense red fluorescence indicating a strong DNA damage. Although there is a slight increase in sperm damage in *Prm2*^+/−^ animals compared to wildtype mice, more than 90% of *Prm2*^+/−^ sperm presented intact (Fig. 3k).

### *Prm2*-deficient sperm are immotile

**Figure 4:**
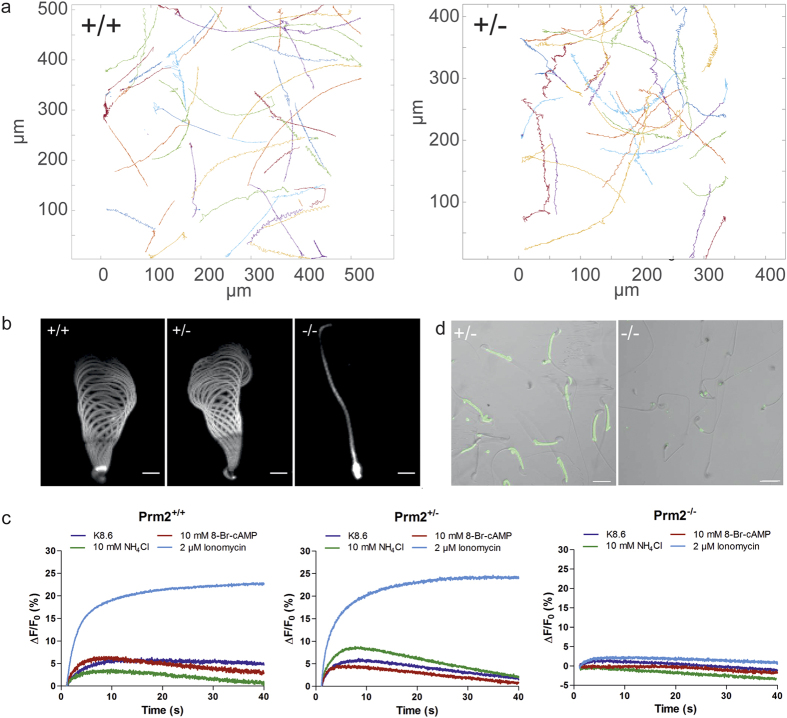
Functional sperm analysis. (**a)** Tracks for freely swimming wildtype *Prm2*^+/+^ and heterozygous *Prm2*^+/−^ sperm. (**b)** Flagellar waveform. Sperm were tethered with their heads to a glass surface and the flagellar waveform was analyzed. One beat cycle was projected. Scale bar: 10 μm. (**c)** Changes in the intracellular Ca^2+^ concentration in *Prm2*^+/+^, *Prm2*^+/−^, and *Prm2*^−/−^ sperm. Sperm have been loaded with CAL520-AM and stimulated with K8.6 (blue), 10 mM 8-Br-cAMP (red), 10 mM NH4Cl (green), or 2 μM ionomycin (light blue). Experiments have been measured using the stopped-flow technique. (**d**) Loading of sperm with Cal520-AM. Loading of *Prm2*^+/−^, and *Prm2*^−/−^ sperm was tested using fluorescence microscopy. Scale bar = 20 μm.

To reveal whether sperm motility in addition to sperm morphology was affected by loss of *Prm2*, we isolated wildtype, heterozygous, and *Prm2*-deficient sperm and analyzed the motility of freely swimming sperm using computer-assisted semen analysis (CASA) and the flagellar waveform of tethered sperm. While motility parameters of freely swimming sperm form wildtype and heterozygous Prm2Δ^97^ or Prm2Δ^101^ animals were not significantly different (Table 2, Fig. 4a), homozygously *Prm2*-deficient sperm were completely immotile (Fig. 4b). Wildtype and heterozygous mice displayed a symmetrical flagellar waveform, which was absent in *Prm2*-deficient sperm (Fig. 4b). Sperm function, in particular sperm motility, crucially relies on Ca^2+^ signaling^37^,^38^,^39^. Thus, we analyzed Ca^2+^ signaling in *Prm2*-deficient mice using the kinetic stopped-flow technique to measure changes in the intracellular Ca^2+^ concentration in sperm populations loaded with the Ca^2+^ dye Cal520-AM. We applied a number of different stimuli to evoke a Ca^2+^ influx in sperm. Stimulation with K8.6, a buffer that depolarizes and alkalizes the cell and thereby opens the principal Ca^2+^-channel in mammalian sperm, CatSper, evoked a Ca^2+^ response in wildtype and *Prm2*-heterozygous, but not in deficient mice (Fig. 4c). Similarly, alkalization of sperm using NH_4_Cl and direct opening of CatSper using 8-Br-cAMP evoked a Ca^2+^ response in wildtype and *Prm2*-heterozygous, but not in deficient sperm (Fig. 4c). As a control, we applied the Ca^2+^-ionophore ionomycin, which resulted in Ca^2+^ influx in controls and *Prm2*-heterozygous, but not in Prm2-deficient sperm (Fig. 4c). To test whether *Prm2*-deficient sperm were loaded with Cal520-AM, we compared *Prm2*-heterozygous and deficient sperm using fluorescence microscopy (Fig. 4d). Heterozygous sperm were loaded with the dye, showing prominent fluorescence in the midpiece, whereas loading of *Prm2*-deficient sperm was negative (Fig. 4d).

Of note, low levels of fluorescence could also indicate that the intracellular Ca^2+^ concentration in *Prm2*-deficient sperm is extremely low. However, this can be excluded since treatment with ionomycin did not evoke a Ca^2+^ response. Together with the results obtained by transmission electron microscopy (TEM), this suggests that *Prm2*-deficient sperm have severe plasma membrane defects, which might prevent dye loading.

### *Prm1* expression is maintained in *Prm2*-deficient mice

**Figure 5:**
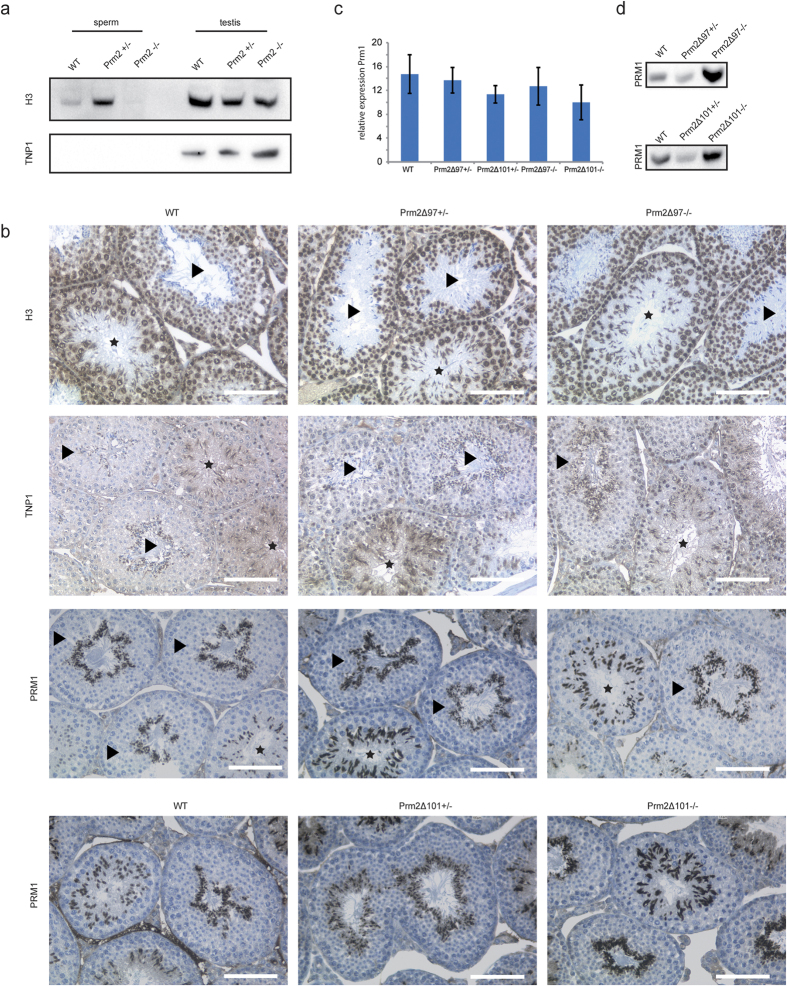
Effects of *Prm2*-deficiency on histone-to-protamine exchange dynamics. (**a**) Immunoblot against H3 and TNP1 of basic nuclear proteins of epididymal sperm and testis from *Prm2*^+/+^, ^+/−^ and ^−/−^ mice **(b)** Immunohistochemical staining of total H3, TNP1 and PRM1 on testicular sections from Prm2Δ mice. Sections were counterstained with hematoxylin. Tubules with elongating spermatids were marked with a star, tubules with mature spermatids with arrowheads. Scale bar = 100 μm. **(c)** Relative expression of *Prm1* mRNA in murine testis of wildtype, *Prm2*^+/−^ and *Prm2*^−/−^ mice measured by qRT-PCR (n = 3). **(d)** Immunoblot against PRM1 following acid urea gel electrophoresis of basic nuclear proteins isolated from epididymal sperm of *Prm2*^+/+^, *Prm2*^+/−^, and *Prm2*^−/−^ mice. Reanalysis of blots shown in Fig. 2e.

Next, we analyzed the effects of *Prm2*-deficiency on the overall histone-to-protamine exchange dynamics. Western blot analysis of basic nuclear proteins isolated from epididymal sperm revealed weak levels of histone H3 in wildtype sperm (Fig. 5a). This is in accordance with previous studies, which described that around 1% of histones remain bound to sperm chromatin after histone-to-protamine exchange^40^. Interestingly, an increased level of H3 was observed in sperm from *Prm2*^+/−^ animals, whereas no H3 was detectable in *Prm2*-deficient sperm (Fig. 5a). No differences in H3 level were observed in protein extracts from testicular tissue (Fig. 5a). IHC stainings gave evidence that the histone replacement is functional. While spermatogonia, round spermatids, and elongating spermatids stained positive for H3, fully elongated spermatids displayed no or only very weak staining (Fig. 5b). Immunoblotting against TNP1 revealed a complete absence in epididymal sperm (Fig. 5a). As a control, presence of TNP1 in testicular tissue was shown. In accordance with this, IHC staining revealed expression of TNP1 in elongating spermatids. In elongated spermatids, where transition proteins are assumed to be completely exchanged by protamines, staining for TNP1 was only observed in residual bodies of *Prm2*^+/+^ and *Prm2*^+/−^ spermatids (Fig. 5b). Interestingly, sections of *Prm2*-deficient animals showed an additional staining within elongated spermatids (Fig. 5b), suggesting a disturbed TNP1 exchange.

The importance of the species-specific ratio of protamines for male fertility suggests that *Prm1* and *2* expression levels are tightly controlled. Therefore, we next analyzed the effects of *Prm2*-deficiency on *Prm1* gene expression. qRT-PCR analysis revealed no significant differences in testicular *Prm1* mRNA levels in males heterozygous or deficient for *Prm2* compared to wildtype controls (Fig. 5c). IHC staining detected PRM1 protein in elongating spermatids of wildtype, heterozygous, and homozygous Prm2Δ^97^ and Prm2Δ^101^ animals (Fig. 5b). In the seminiferous epithelial cycle, cells of step 10 were the first cells expressing *PRM1*. Signal intensity was highest in step 12–15 elongated spermatids, marking the replacement of transition proteins by protamines. Western blot analysis of basic nuclear proteins isolated from epididymal sperm further demonstrated the successful incorporation of PRM1 into sperm chromatin (Fig. 5d). Interestingly, PRM1 protein level were slightly decreased in *Prm2*^+/−^ animals, but increased in *Prm2*-deficient sperm compared to controls (Fig. 5d). Of note, for western blot analysis the basic proteins like the protamines were isolated from precipitated DNA. The fact that we could detect PRM1 in PRM2-deficient sperm suggests that PRM1 is incorporated into sperm chromatin independent of PRM2.

## Discussion

In the present study, we used CRISPR/Cas9 gene-editing to generate *Prm2*-deficient mice to investigate the role of PRM2 in controlling chromatin hypercondensation and fertility. We demonstrate that *Prm2*-heterozygous mice remain fertile with sperm being morphologically and functionally indistinguishable from wildtype sperm. However, lack of *Prm2* causes infertility with severe defects in sperm head morphology and sperm motility.

Although the role of protamines for sperm function has been described many times for different species^41^, our observation that loss of one *Prm2* allele does not affect male fertility in mice is contradictory to previous studies. Cho *et al*. described that already chimeras produced by injecting *Prm2*^+/−^ ES cells were sterile^29^. We hypothesize that these fundamental differences between our study and the observations made by Cho *et al*. might be due to the different experimental approaches used to generate *Prm2*-knockout alleles. Cho *et al*. applied a classical gene-targeting approach in ES cells to disrupt the *Prm2* coding region by insertion of a Pgk-neo cassette^29^. This cassette might have affected the expression in the tightly regulated 11.7 kb spanning gene cluster encoding for *Prm1*-*Prm2*-*Tnp2*^12^. Insertion of the Pgk-neo cassette into the *Prm2* coding region might have resulted in silencing of the neighboring *Prm1* gene. In fact, such a trans-silencing has been described for the knockout of the herculin (*Myf6*) gene. Here, the Pgk-neo cassette introduced by homologous recombination to disrupt the *Myf6* locus silenced expression of the nearby myogenic factor 5 (*Myf5*) gene^42^,^43^. We propose that the trans-silencing of the *Prm1* allele may be caused by the Pgk-neo cassette inserted into the *Prm2* allele. This assumption is supported by the fact that testes of chimeric mice generated by injecting *Prm2*^+/−^ ES cells displayed a strong reduction in PRM1 protein level^29^. In our model, qRT-PCR clearly demonstrates that expression of *Prm1* is maintained in *Prm2*^+/−^ males, while only a mild decrease in protein level is observed.

Further, the hypothesis that physiological PRM2 protein levels are required for proper incorporation of PRM1 into sperm chromatin has to be re-visited^29^, since sperm of *Prm2*^+/−^ and *Prm2*^−/−^ males displays certain degrees of DNA-hypercondensation indicative for DNA-protamine interaction.

Here, we utilized the CRISPR/Cas9 system to generate *Prm2*-deficient mice. This state-of-the-art technique allows to efficiently edit genes without introducing additional DNA elements like loxP sites and/or antibiotic resistance cassettes^31^. Off-target activity is a highly discussed issue in the field of CRISPR/Cas9-mediated genome editing. Nonetheless, different approaches analyzing potential off-target effects in genetically altered mice and ES cells, which closely resemble the situation in zygotes, showed either no or only a very low incidence for CRISPR/Cas9-mediated off-target activity^44^,^45^, when compared with analyses carried out (mainly) in cancer cell lines, which are hallmarked by genomic instability^46^,^47^,^48^. To reduce the risk for off-target events, we selected only gRNAs with very low predicted off-target activity. Furthermore, if possible we used 18-mer guide sequences instead of 20-mer as this was shown to further decrease the frequency of Cas9-mediated off-target events^49^. In addition, the Prm2Δ^97^ and Prm2Δ^101^ alleles were backcrossed with wildtype mice. Thus, potential off-target effects, which might have affected loci on other chromosomes, are lost during further breeding.

We established two mouse lines, Prm2Δ^97^ and Prm2Δ^101^, with N-terminal deletions of 97 and 101 bp, respectively. Both mutations did not interfere with or deleted regulatory regions and left the overall structure of the gene cluster intact. Male mice heterozygous for the respective mutation were fertile and sired offspring with an average litter size of 8.6, which is within the range of the reproductive performance of wildtype mice with a mean litter size of 6.2 and a maximum mean litter size of 9.5 in the plateau period of reproduction^34^,^35^. This clearly indicates that deletion of one *Prm2* allele does not affect male reproductive performance in mice. The typical apical hook-like structure of sperm heads and intact sperm motility further suggests that sperm physiology and morphology are not affected.

This raises the question whether disturbed chromatin integrity or the loss of sperm motility and detachment of the acrosome is the main reason for infertility of knockout mice. The latter is supported by Takeda *et al*., who were able to generate offspring from *Prm1*^+/−^ males by *in vitro* fertilization of zona-free oocytes, although chromatin integrity of those sperm was severely affected^50^. Nontheless, strong sperm DNA degradation observed in *Prm2*-deficient animals most likely prohibits successful reproduction.

Protamines are expressed in haploid spermatids, hence in *Prm2*^+/−^ mice, only half of the spermatids express *Prm2*. However, IHC staining revealed PRM2 protein in all spermatids of seminiferous tubules. While transcript or protein sharing among syncytial spermatids has been discussed in general, the IHC stainings shown here clearly demonstrate that such sharing exists for *Prm2*^29^,^51^,^52^,^53^.

Of note, knockout male mice heterozygous for the transition proteins TNP1 and TNP2 display preserved fertility as well, while complete deletion of both alleles resulted in subfertility^54^,^55^.

*Prm2*-heterozygous mice display reduced levels of PRM2 protein, compared to controls, and show maintained expression of PRM1. We demonstrate that these mice sire normal litter sizes indicative of full fertility. Western blot showed that in *Prm2*-heterozygous mice deletion of one allele of *Prm2* not only led to a reduction of PRM2 protein but also caused a moderate decrease of PRM1 protein level. This is suggestive for a PRM1/2 coregulation in order to maintain the mouse specific PRM1/2 ratio. Recent analyses revealed that PRM1/PRM2 ratio range from 65% (*M. castaneus* and *M. domesticus*) to 99% (*M. macedonicus* and *M. spicilegus*) in different mouse subspecies^56^. This indicates that mice might be able to tolerate changes in the PRM1/2 ratio to some extent. This would be in contrast to the situation in humans, where changes of the PRM1/2 ratio are associated with reduced fertility. Further, the elevated levels of histones detected in such animals that remain bound to sperm chromatin might compensate for the lower total amount of protamines.

*Prm2*-deficient sperm displayed upregulation of PRM1. We hypothesize that the higher PRM1 levels are an attempt to compensate for the loss of PRM2. A similar compensatory effect has been described for *Tnp2*-deficient mice, which showed enhanced *Tnp1*-expression^55^. Sperm from *Prm2*^−/−^ animals lost the characteristic hook-like structure: sperm exhibited round heads and were immotile. Interestingly, a recent study suggests that the cleaved N-terminal part of PRM2 rather than the chromatin-bound mature PRM2 is involved in controlling sperm head morphology^57^. However, the underlying molecular mechanism is not known. In TEM analysis, we were able to show a detachment of the acrosomal cap and a less-condensed chromatin. Similar observations were made for protamine-deficient human sperm^58^. Whereas the reason for the defect in chromatin packaging is obvious, the molecular mechanisms underlying the ultrastructural change in acrosomal cap formation and attachment to the nucleus need to be addressed in further studies.

But why are sperm from *Prm2*-deficient mice immotile? Once histones are replaced by protamines, transcription is silenced^6^. Thus, transcriptional regulation of other genes by *Prm2* seems unlikely. Further, in *Prm2*-deficient males, development of testicular spermatids is not affected and sperm counts are comparable. Profound morphological aberrations in *Prm2*-deficient mice are first apparent in the epididymis with detachment of acrosome and bending of the flagellum towards the head. In parallel, treatment with Ca^2+^ ionophores indicates that epididymis derived *Prm2*-deficient sperm presents with defects in membrane function. It remains to be determined whether this is a defect in the self-assembly of sperm or a built-in quality control mechanism triggering a self-destructing program. Of note, impaired sperm motility is a common phenotype of *Prm1, Prm2, Tnp1*, and *Tnp2* knockout mice^29^,^50^,^54^,^55^,^59^.

With Prm2Δ^97^ and Prm2Δ^101^ mouse lines, we have established, for the first time, a robust and reliable model for functional studies on protamine-induced chromatin hypercondensation during spermiogenesis. These mice allow for a detailed investigation of basic regulatory mechanisms of haploid gene expression in sperm. Shedding light on these molecular mechanisms is an absolute requirement for a better understanding of aberrant protamine expression in subfertile men.

## Material and Methods

### Ethics statement

All animal experiments were conducted according to German law of animal protection and in agreement with the approval of the local institutional animal care committees (Landesamt für Natur, Umwelt und Verbraucherschutz, North Rhine-Westphalia, approval ID: AZ84-02.04.2013.A429).

### Guide RNA design and cloning

Gene-specific guide sequences (gRNAs) (Supplementary Table 1) were designed using the CRISPR design tool (http://crispr.mit.edu)^60^ and inserted into the pX330 expression vector (pX330-U6-Chimeric_BB-CBh-hSpCas9 Addgene plasmid # 42230))^61^ as described previously^62^. Some gRNAs were truncated to 19- or 18-mers, to ensure initial guanine or adenine bases and to reduce potential off-target activity^49^.

### Culture and transfection of mES cells

E14Tg2a mES cells (kind gift of Christof Niehrs, IMB Mainz, Germany) were maintained on gelatinized cell culture dishes with standard ES cell medium at 37 °C and 7.5% CO_2_. For transfection, 3 × 10^5^ cells per well were seeded onto a gelatinized 12-well-plate in media without antibiotics. After 3 h cells were transfected with a 3:1 ratio of pX330: Lipofectamine2000, according to the manufacturers’ protocol (Thermo Fisher Scientific, Waltham, USA). 8 h later media was changed to ES media to remove DNA-Lipofectamine complexes.

### Surveyor assay

Two days after transfection, genomic DNA was extracted from cells using the phenol-chloroform-isoamyl-alcohol (PCI) precipitation method as described previously^63^. Gene edited loci were amplified by PCR (see Supplementary Table 1 for primer sequences). Products were denaturated by heating to 95 °C for 10 min, re-annealed by stepwise cooling to room temperature (RT), and analyzed using the ‘SURVEYOR Mutation Detection Kit’ (Transgenomic, Manchester, GB).

### Generation of Prm2-deficient mice

Female B6D2F1 mice were superovulated by intraperitoneal injection of pregnant mare’s serum (PMS, 5 i.u.) and human chorionic gonadotropin (hCG, 5 i.u.) and mated 2:1 with B6D2F1 males. Zygotes were isolated at 0.5 dpc from the oviducts and subjected to microinjection using an inverted microscope (Leica, Wetzlar, Germany) equipped with micromanipulators (Narishige, Japan) and piezo unit (Eppendorf, Hamburg, Germany). Injection pipettes (PIEZO 8-15-NS, Origio, Charlottesville, USA) were filled with Fluorinert (FC-770, Sigma-Aldrich, Taufkirchen, Germany) for appropriate piezo pulse propagation. *In vitro* transcribed single-guide RNAs (sgRNA) (50 ng/μl each) and *Cas9* mRNA (100 ng/μl) (Sigma-Aldrich) were injected into the cytoplasm of zygotes, as described previously^31^. Surviving zygotes were cultured in KSOM medium for 3 days. Developing blastocysts were transferred into the uteri of pseudo-pregnant foster mice.

### Quantitative reverse transcription–polymerase chain reaction (qRT-PCR)

RNA was isolated from testis tissue with TRIzol (Life Technologies, Carlsbad, USA). RNA concentrations and purity ratios were determined by NanoDrop measurement (Peqlab, Erlangen, Germany). qRT-PCR was performed as described previously^64^. First strand cDNA synthesis was carried out using RevertAid First Strand cDNA Synthesis Kit (Fermentas, St. Leon-Rot, Germany) after DNAseI treatment of RNA. qRT-PCR was performed on ViiA 7 Real Time PCR System (Applied Biosystems, distributed by Life Technologies) using Maxima SYBR Green qPCR Master Mix (Life Technologies). At the end of each PCR run, a melting point analysis was performed. *Actin-beta* was used as reference gene for data normalization. See Supplementary Table 1 for primer sequences.

### Antibodies

Mouse monoclonal antibodies anti-PRM1 (Hup1N) and anti-PRM2 (Hup2B) (Briar Patch Biosciences, Livermore, CA, USA, IB 1:2.000, IHC 1:100), rabbit anti-H3 (Abcam, Ab1791, IB 1:2.000, IHC 1:400) and rabbit anti-TNP1 (Abcam, Ab73135, IB 1:1.000, IHC 1:400) were used in this study. Secondary polyclonal antibodies were used as follows: rabbit anti-mouse HRP 1:1.000 (Dako, Hamburg, Germany), goat anti-rabbit HRP 1:2000 (Dako), goat anti-mouse BIOT 1:200 (Dako).

### Isolation of epididymal sperm

Sperm were isolated by multiple incisions of the cauda followed by a swim-out in modified TYH medium^65^ or M2 medium. Sperm count was determined using a Neubauer hemocytometer. For all experiments, sexually mature males with an age of 2–5 months were used. Animals were derived from intercrosses of the F1 generation.

### Isolation of sperm nuclear proteins

Nine million sperm were washed with phosphate-buffered saline (PBS, pH 7.4) containing protease inhibitors (Complete mini, Roche, Basel). Extraction of basic nuclear proteins was performed according to Deyebra and Oliva^66^.

### Immunoblotting

Protein extracts were subjected to 15% polyacrylamide acid-urea gel electrophoresis^67^,^68^ and blotted onto a polyvinylidene fluoride (PVDF) membrane using the Trans-Blot Turbo system (BioRad, Munich, Germany). Equal protein loading was validated by staining with Coomassie Brillant Blue G-250. Membranes were blocked in 3% nonfat dry milk powder in Tris-buffered saline with Tween20 (TBST) for 2 h at RT and probed with primary (overnight at 4 °C) and secondary (1 h at RT) antibodies in blocking solution. Chemiluminescent signals were detected using ChemiDoc MP Imaging System (BioRad) after incubation of the membrane with PierceSuper Signal West Pico chemiluminescent substrate (Perbio, Bonn, Germany) or Westar Supernova substrate (7Bioscience, Hardtheim, Germany).

### Immunohistochemistry

Bouin’s fixed testicular tissue was processed in paraffin wax. Immunohistochemistry (IHC) against PRM1 was performed as described previously^64^ using the Autostainer 480 S (Medac, Hamburg, Germany). For IHC of PRM2, sections were treated for 10 min at RT with decondensing-mix containing 25 mM dithiothreitol, 0.2% Triton X-100 and 200 IU heparin/ml in PBS to enhance antigen accessibility for the primary antibody^69^. Subsequently, sections were washed in 0.02 M PBS (pH 7.4), boiled for 20 min in sodium citrate buffer, treated for 20 min with 3% H_2_O_2_ in methanol and blocked for 20 min with 5% bovine serum albumin (BSA) in PBS. Incubation with the primary antibody was performed overnight at 4 °C, with biotinylated goat-anti-mouse secondary antibody (DAKO) and the Vectastain ABC Kit (Vector Laboratories, Burlingame, CA, USA) for 1 h at RT each. Immunoreaction was visualized using AEC (DAKO). Finally, sections were counterstained with hematoxylin and covered with glycerin gelatin.

### Electron microscopy

Testis and epididymis perfused with PBS buffer (0.1 M) including 0.25% heparin followed by Yellow-Fix (2% paraformaldehyde, 0.1 M sodium cacodylate buffer pH 7.2, 0.02% picric acid mixed with 2% glutaraldehyde just before usage) via the heart were immersion-fixed in Yellow-Fix for 24 h at 4 °C. Smaller samples rinsed in PBS buffer (0.1 M) were then immersion-fixed in 1% osmium tetroxide at 4 °C for 2 h and rinsed in buffer again. Subsequently, the tissue was dehydrated and embedded in Epon. Ultrathin-sections were picked up on grids, stained with lead-citrate (0.2%) and examined with a Zeiss EM 109 (Zeiss, Oberkochen, Germany).

### Assessment of sperm DNA damage

For isolation of sperm genomic DNA, ten million sperm were resuspended in 500 μl lysis buffer (10 mM Tris HCl, 25 mM EDTA, 1% SDS, 75 mM NaCl) and supplemented with 21 μl 1 M DTT, 2,5 μl Triton x-100 and 40 μl Proteinase K (10 mg/ml). The suspension was incubated at 55 °C for 2 h following PCI precipitation of DNA. SCSA-like analysis was performed referring to Evenson *et al*.^70^.

### Measurement of changes in sperm intracellular Ca^2+^

Sperm were isolated by incision of the cauda followed by a swim-out in modified TYH medium^65^. Sperm were loaded with the fluorescent Ca^2+^ indicator Cal-520, AM (AAT Bioquest, Sunnyvale, USA) (5 μM) in the presence of Pluronic F-127 (0.05% vol/vol) at 37 °C for 45 min. After incubation, excess dye was removed by three centrifugation steps (700 × g, 7 min, RT). The pellet was resuspended in TYH and equilibrated for 5 min at 37 °C. Pictures of loaded sperm were taken on a confocal microscope (FV1000; Olympus). Changes in [Ca^2+^]_i_ were recorded in a rapid-mixing device in the stopped-flow mode (SFM400; Bio-Logic, Claix, France) as previously described^65^. Data acquisition was performed with a data acquisition pad (PCI-6221; National Instruments, Austin, USA) and Bio-Kine software v. 4.49 (Bio-Logic). Ca^2+^ signals are depicted as the percent change in fluorescence (ΔF) with respect to the mean of the first three data points recorded immediately after mixing (F_0_), that is, when a stable fluorescence signal was observed. The control (buffer) ΔF/F_0_ signal was subtracted from compound-induced signals.

### Immunocytochemistry

Sperm were dried and fixed in 4% paraformaldehyde/PBS for 20 min. To block unspecific binding sites, sperm were incubated for 40 min with blocking buffer (0.5% Triton X-100 and 5% ChemiBLOCKER (Merck Millipore, Darmstadt, Germany) in 0.1 M phosphate buffer, pH 7.4. PSA-FITC (0.5 mg/ml, Sigma Aldrich) and MitoTracker Red CMXRos (1:2000, Thermo Fisher) were diluted in blocking buffer containing 0.5 mg/ml DAPI (Life Technologies) and incubated for 1 h. Pictures were taken on a confocal microscope (FV1000; Olympus).

### Motility analyses

Sperm motility was studied in shallow observation chambers (depth 150 μm) using an inverted microscope (IX71; Olympus) equipped as described previously^65^.

To analyze the flagellar beat, cells were tethered to the glass surface by adjusting the BSA concentration in the buffer from 3 to 0.3 mg/ml. Cells that had their head attached to the glass surface and had a free beating flagellum were selected for imaging. Images were collected at 200 frames per second using a CMOS camera (Dimax; PCO, Kelheim, Germany). Quantification of the flagellar beat was performed using custom-made programs written in Java, which can be made available upon request. The program identified a point on the flagellum 25 μm apart from the center of the mouse head within every time frame. The flagellar beat parameters were determined within a time window of 1 s after each frame. The first point in this time window was chosen as reference point and the distance between the successive points found on the flagellum and the reference point was monitored. This distance varied in a sinusoid-like manner in time and the beat frequency was determined as the maxima in the Fourier spectrum.

For analyzing the swimming parameters of sperm cells, the BSA concentration in the buffer was 3 mg/ml, resulting in most of the cells swim freely at the glass surface. Images were collected at 50 frames per second (Dimax). Quantification of the swimming parameters was performed the Casa program^71^ adapted to MATLAB (MathWorks. Natick, USA).

### Statistics

For mating experiments, sperm count analysis, testis weight, qRT-PCR, and FACS analysis mean values are shown. Error bars represent standard deviation (SD). Statistical significance was determined by two-tailed, unpaired Student’s t-test. Significance was assumed for p-values < 0.05 (*p < 0.05; **p < 0.005; ***p < 0.001).

## Additional Information

**How to cite this article**: Schneider, S. *et al*. Re-visiting the Protamine-2 locus: deletion, but not haploinsufficiency, renders male mice infertile. *Sci. Rep.* **6**, 36764; doi: 10.1038/srep36764 (2016).

**Publisher’s note:** Springer Nature remains neutral with regard to jurisdictional claims in published maps and institutional affiliations.

## Supplementary Material

Supplementary Information

## Figures and Tables

**Table 1:** Injection statistics.

#ID	Zygotes injected	Intact after injection (% of injected)	Blastocysts transferred (% of intact)	Prm2 edited/pups born (%)
A	150	108 (72.0)	21 (19.4)	1/3 (33.3)
B	186	137 (73.7)	61 (44.5)	3/7 (42.9)
total	336	245 (72.9)	82 (33.5)	4/10 (40.0)

Efficiency of CRISPR/Cas9 microinjections displayed as survival rates of oocytes and number of modified alleles obtained to total pups born.

**Table 2:** Motility parameters.

genotype	VCL	VAP	VSL	LHD	STR
wildtype	100 ± 43	49 ± 18	34 ± 22	2.2 ± 1.2	67 ± 30
Prm2 Δ97^+/−^	69 ± 25	45 ± 21	21 ± 14	2.1 ± 1.1	46 ± 29
Prm2 Δ101^+/−^	97 ± 49	62 ± 25	32 ± 22	2.4 ± 1.5	53 ± 32

Free-swimming sperm from wildtype or heterozygous Prm2Δ 97 or Prm2Δ 101 mice have been analyzed using dark-field microscopy. Data are presented as mean ± S.D.; VCL: curvilinear velocity μm/s), VAP: average path velocity (μm/s); VSL: straight-line velocity (μm/s), LHD: lateral head displacement (μm); STR: path straightness (VSL/VAP).
