# Supplementary material for: Re-visiting the Protamine-2 locus: deletion, but not haploinsufficiency, renders male mice infertile
Source: Sci Rep. 2016 Nov 11;6:36764. doi: 10.1038/srep36764 (PMC5105070; doi:10.1038/srep36764)
Supplement: Supplementary Information [file srep36764-s1.pdf]

Re-visiting the Protamine-2 locus: deletion, but not haploinsufficiency, renders male mice infertile

Simon Schneider[1], Melanie Balbach[2], Jan Jikeli[2], Daniela Fietz[3], Daniel Nettersheim[1], Sina Jostes[1], Rovenna Schmidt[3], Monika Kressin[3], Martin Bergmann[3], Dagmar Wachten[2], Klaus Steger[4] and Hubert Schorle[1]

[1]Institute of Pathology, Department of Developmental Pathology, University of Bonn Medical School, Bonn, Germany
[2]Minerva Max Planck Research Group - Molecular Physiology, Center of Advanced European Studies and Research, Bonn, Germany
[3]Institute of Veterinary-Anatomy, -Histology and -Embryology, Justus-Liebig University, Giessen, Germany
[4]Department of Urology, Pediatric Urology and Andrology, Section Molecular Andrology, Biomedical Research Center of the Justus-Liebig University, Giessen, Germany

*Correspondence to Prof. Dr. Hubert Schorle, Institute of Pathology, Department of Developmental Pathology, University Hospital Bonn, Sigmund-Freud-Str. 25, 53127 Bonn, Germany.

E-mail: Hubert.Schorle@ukb.uni-bonn.de

Supplementary Table 1: Oligonucleotides used in this study.

Oligonucleotides used for cloning and *in vitro* transcription of gRNAs, genotyping and qRT-PCR.

guide sequences	top strand oligo	bottom strand oligo
Prm2 gRNA1	CACCATGGTTCGCTACCGAATG	AAACCATTCGGTAGCGAACCAT
Prm2 gRNA2	CACCATAGTCCTCTACGCGCTC	AAACGAGCGCGTAGAGGACTAT
Prm2 gRNA3	CACCGCTGAGCCCAGAGCGCGTAG	AAACCTACGCGCTCTGGGCTCAGC

PCR primer	forward primer	reverse primer
Prm2 sg1	CCCCAAACACCAGACCATCA	CCCTGTGTGTCCTCCCATAG
Prm2 sg2/3	GGTTCGCTACCGAATGAGGA	GGGTGCTTACCTCTGCGAT
Prm2 sg1-3	TGCAGCCTCAATCCAGAACC	TGTAGCCTCTTACGAGAGCAG

in vitro transcription	forward primer	reverse primer
T7-Prm2-sgRNA1	TTAATACGACTCACTATAGGATGGTTCGCTACCGAATG	-
T7-Prm2-sgRNA2	TTAATACGACTCACTATAGGATAGTCCTCTACGCGCTC	-
T7-Prm2-sgRNA3	TTAATACGACTCACTATAGGGACAAGACCATGAACGCG	-
T7-sgRNA	-	AAAAGCACCGACTCGGTGCCGCTGAGCCCAGAGCGCGTAG

qRT-PCR	forward primer	reverse primer
Prm2	ATGGTTCGCTACCGAATGAGG	CTCCGCCTTCTGCATGACC
Prm1	GACAGCCCACAAAATTCCAC	CAGAGCAGGGGACACCAC
beta-Actin	TGTTACCAACTGGGACGACA	GGGGTGTTGAAGGTCTCAAA
